# Tea ( *Camellia sinensis* ) Seed Saponins Act as Sebosuppression Agents via the AMPK/mTOR Pathway

**DOI:** 10.1111/jocd.16793

**Published:** 2025-01-21

**Authors:** Jian Li, Yuan‐cheng Huang, Jian‐ming Deng, Min Yu, Christos C. Zouboulis, Guang‐Li Wang, Jing Wang

**Affiliations:** ^1^ Cosmetic Research Center, School of Chemical and Material Engineering Jiangnan University Wuxi China; ^2^ Guangzhou Huashi Cosmetic Technology Co. Ltd. Guangzhou Guangdong China; ^3^ Department of Dermatology, Venereology, Allergology and Immunology, Staedtisches Klinikum Dessau Brandenburg Medical School Theodore Fontane and Faculty of Health Sciences Dessau Germany

**Keywords:** AMPK/mTOR, human sebocytes, lipid synthesis, tea seed saponins

## Abstract

**Background:**

Excessive lipogenesis of the skin triggers some dermatological concerns, such as enlarged pores, acne, and blackheads. Although topical drug treatments can offer temporary relief, their prolonged usage may lead to side effects of dryness, irritation, or allergic reactions. Consequently, the development of safer and efficacious ingredients in cosmetics for managing sebum overproduction represents a significant yet challenging endeavor.

**Aim:**

Saponins were extracted from tea ( *Camellia sinensis* ) seed meal and purified by macroporous resin in order to investigate the impact of tea seed saponins (TSS) on lipid production in human immortalized sebaceous cells. Moreover, we attempted to reveal the underlying mechanism of TSS on the sebosuppression effect in SZ95 sebocytes stimulated by linoleic acid (LA).

**Methods:**

The compositions and chemical structures of TSS were determined using UV–vis absorption spectrum, Fourier transform‐infrared (FTIR) spectrum, and ultra‐high‐performance liquid chromatography‐mass spectrometry analysis. An in vitro model of cellular lipid accumulation induced by LA was established. Total lipid synthesis in intracellular SZ95 sebocytes was assessed through Nile Red staining, while triglyceride, cholesterol, and fatty acids were quantified by commercially assay kits. Western blot and quantitative real‐time polymerase chain reaction were employed to analyze the protein expression levels involved in the AMP‐activated protein kinase (AMPK)/mammalian target of rapamycin (mTOR) pathway as well as the downstream protein and mRNA expressions of sterol regulatory element‐binding protein‐1 (SREBP‐1), peroxisome proliferator‐activated receptor γ (PPARγ), and fatty acid synthase (FAS). The localizations of SREBP‐1 within the cytoplasm or nucleus were characterized using immunofluorescence staining.

**Results:**

Five saponins were identified in the extracted TSS, all of which were oleanic acid‐type pentacyclic triterpenes. TSS treatment significantly alleviated LA‐induced lipid accumulation in SZ95 sebocytes. In addition, TSS activated the AMPK/mTOR pathway and downregulated the downstream protein and mRNA expression of transcription factors and enzymes, including SREBP‐1, PPARγ, and FAS. Moreover, the TSS blocked the nuclear transfer of SREBP‐1 from cytoplasm to nucleus.

**Conclusion:**

In human sebocytes, TSS exhibited sebosuppressive effect as revealed by the inhibited production of total lipids as well as triglyceride, cholesterol, and fatty acids. Moreover, the anti‐lipogenesis mechanism by TSS involved the activation of the AMPK/mTOR pathway and downregulated downstream transcription factors and enzymes of SREBP‐1, PPARγ, and FAS. Additionally, TSS blocked the SREBP‐1 nuclear translocation. These results may justify the potent of TSS as a new candidate for modulating lipogenesis in human SZ95 sebocytes.

**Abbreviations**
LA: linoleic acid
SZ95: human sebaceous gland cell line (SZ95)
TSS: tea seed saponin

## 1 Introduction

Skin sebum, secreted by cells of the sebaceous gland, mainly contains diverse neutral lipids [1]. It enhances skin barrier by virtue of its prevention for water loss and resistance against invasion from externally harmful species [2]. However, excessive secretion of sebum is prone to cause lipid accumulation and then result in abnormal production of microorganisms and lipid peroxidation, which ultimately lead to annoying skin problems such as pore blockage, blackheads, and acne [3, 4, 5]. There are different pathways, including the dihydrotestosterone (DHT) modulated androgen receptor (AR) pathway [6], insulin‐like growth factor 1 receptor activated phosphatidylinositol‐3‐kinase/protein kinase B and mitogen‐activated protein kinase/extracellular signal‐regulated kinase pathways [7, 8], and AMP‐activated protein kinase (AMPK)/mammalian target of rapamycin (mTOR) pathway [9, 10, 11], and so forth, that can regulate the lipid synthesis of sebaceous gland cells. Among which, the AMPK/mTOR pathway is a key pathway that regulates lipid production through its downstream transcription factors of sterol regulatory element‐binding protein‐1 (SREBP‐1) and peroxisome proliferator‐activated receptor gamma (PPARγ) [12, 13]. And fatty acid synthase (FAS), as a target gene of SREBP‐l, plays a pivotal role in fatty acid synthesis [14]. Topically adopted active drugs (e.g., isotretinoin, benzoyl peroxide combined with antibiotics, benzoyl peroxide combined with topical retinoids) [15, 16] are effective in inhibiting superfluous secretion of sebum, but their serious side effects, including dryness and irritation hinder their wide and long‐term usage. The exploration of newly active ingredients in cosmetics to treat sebum hypersecretion, which are safer but still with high efficacy, has been a hot pursuit in both academic research and industrial community.

Botanic extracts are popular as active ingredients in cosmetic applications for skin health promotion. Saponins, a class of active ingredients extracted from plants, have gained widespread popularity in cosmetics as nonirritant cleansing, foaming, and emulsifying agents [17, 18, 19]. Moreover, some progresses [20, 21, 22] have been made for the anti‐oxidant and anti‐inflammatory efficacy of saponins from tea seed. Bearing in mind the prospect of saponins with promisingly wider applications, we aimed to explore the sebum modulation efficacy to further enrich their application scopes.

In this study, we extracted and purified saponins from tea ( *Camellia sinensis* ) seed meal. The structure of the tea seed saponins (TSS) were identified, and the lipogenesis modulation effect as well as the underlying mechanisms of TSS in exerting sebosuppression for linoleic acid (LA)‐stimulated SZ95 sebocytes were revealed. Results suggested that TSS regulated lipid metabolism may potentially reduce lipid accumulation through modulating the AMPK/mTOR pathway.

## 2 Materials and Methods

### 2.1 Extraction and Purification of TSS From Tea Seed Meal

#### 2.1.1 Extraction of TSS

The tea seed meal and 70% ethanol (with the solid to liquid mass ratio of 1:5) were mixed and stirred at 60°C for 4 h. After that, the extract solution was filtered (by a 0.22 μm filter membrane) for filtrate collection until a viscous solution was obtained. In addition, the viscous solution was then washed three with petroleum ether to remove the oil‐soluble substances. Finally, *n*‐butanol was added to the liquid phase to extract the saponins until the extract phase became clear. After collecting the extracts through centrifugation, they were concentrated under reduced pressure until a viscous solution was obtained.

#### 2.1.2 Isolation and Purification of TSS

The crude saponins solution obtained above was further purified by a macroporous resin‐based column chromatography, by referring to the method of Yu et al. [23] with modifications. Specifically, the viscous solution was initially diluted with deionized water and then adsorbed by a D101 macroporous resin, in which the mass ratio of the D101 resin to the saponins solution was around 1:10. After the resin adsorbed the extract, it was sequentially eluted with deionized water, 0.2% NaOH solution, deionized water, and 20% ethanol. Finally, 80% ethanol eluting was carried out, followed by concentrating and freeze‐drying to obtain the purified TSS powder.

#### 2.1.3 Structure and Identification of Saponins From Tea Seed Meal

The prepared TSS sample (10 mg) was dissolved in 20 mL of 80% ethanol and scanned for UV–vis absorption characteristics at 200–400 nm on a Genesys 10S UV–VIS spectrophotometer (Thermo Fisher Scientific, Waltham, MA, USA). The TSS powder was mixed with KBr crystals, then ground and pressed to perform Fourier transform‐infrared (FTIR) spectral analysis (Nicolet iS50 FTIR spectroscopy, Thermo Fisher Scientific) of the chemical structure of TSS.

An ultra‐high‐performance liquid chromatography (UHPLC, AB SCIEX, USA) coupled with a X500R high‐resolution mass spectrometry (MS) system (AB SCIEX, USA) and an OS data acquisition software (AB SCIEX, USA) were used to reveal the detailed structure of the TSS.

Conditions for liquid chromatographic analysis: Waters ACQUITY UPLC BEH C18 column (1.7 μm, 2.1 × 100 mm). Column temperature: 50°C; injection volume: 4.0 μL; mobile phase A: 0.1% formic acid/water; mobile phase B: 0.1% formic acid/acetonitrile; flow rate: 0.3 mL/min. Gradient elution procedure: the initial mobile phase contained 5% B phase and was maintained for 0–2 min; then, the mobile B phase was changed from 5% to 99% during 2–10 min; during the next 10–20 min, the mobile B phase was maintained at 100%; in the final 20–23 min, the mobile B phase was again kept at 5%.

Mass spectrometry analysis conditions: Source temperature 550°C, gas curtain gas flow rate was 35 psi, clustering potential (DP) of 80 V, MS Collision Energy (CE) of 10 eV, MSMS Mode CE was 40 ± 15 eV. The scanning ranges of primary and secondary mass spectrometries were 70–1500 and 50–1500 Da, respectively.

### 2.2 Cell Culture

The human immortalized sebaceous gland cell line SZ95 [24] was cultured in DMEM medium composed of 10% fetal bovine serum (Gibco, Carlsbad, CA, USA), 1.0 × 10^5^ U/L of penicillin and 100 mg/L of streptomycin at 37°C, 5% CO_2_ under humidity saturation. Subcultured cells were treated trypsin containing 0.25% EDTA (Gibco) and were propagated in culture medium as described above.

### 2.3 3‐(4,5‐Dimethylthiaxolone‐2‐yl)‐2,5‐Diphenyl Tetra‐Zoliumbromide Assay for Cell Viability

The cytotoxicity of different substances (TSS, LA, and DHT) was investigated through the 3‐(4,5‐dimethylthiaxolone‐2‐yl)‐2,5‐diphenyl tetra‐zoliumbromide (MTT) assay. To prepare the experimental solutions, a stock solution of TSS (1.0 mg/mL, obtained by dissolving 10 mg of TSS in 10 mL of DMEM); LA (50 mg/mL, obtained by dissolving 100 mg of LA in 2 mL of dimethyl sulfoxide [DMSO]); and DHT (100 mg/mL, obtained by dissolving 100 mg DHT in 1.0 mL of DMSO) were serially diluted with DMEM to achieve desirable concentrations.

The SZ95 sebocytes with a density of 1 × 10^5^ cells/mL were seeded into 96‐well plates with volume of 100 μL per well. After 24 h of culture, 100 μL of different (TSS, or LA, or DHT, or the mixture of TSS and LA, or the mixture of TSS and DHT) solutions at different concentrations (prepared as previously described) were added to each well, and a control group was set up through the addition of DMEM in double six plicate wells. After incubated for 48 h, the MTT (Sigma‐Aldrich Co., Saint Louis, MO, USA) (100 μL, 0.5 mg/mL) was added and incubated for another 4 h. After the above incubation medium was gently removed, 100 μL of DMSO (Sinopharm Group Chemical Reagents Co. Ltd., Beijing, China) was added to dissolve the formazan crystals. The absorbance at 490 nm was measured with a microplate reader (Tecan Infinite 200Pro). Cell viability was calculated by Formula (1‐1).

(1‐1) $$\text{Cell viability}\;\left(\%\right)=\left({\mathrm{OD}}_{T}/{\mathrm{OD}}_{B}\right)\times 100\%$$

where OD_T_ and OD_B_ represent the average OD values of the experimental group and the control group at 490 nm, respectively.

### 2.4 Nile Red Staining and Lipid Content Determination

For the Nile Red staining groups treated with LA or a mixture of LA and TSS, the SZ95 cells were treated with 100 μL of LA at a concentration of 10.0 μg/mL or with mixtures of TSS and LA (10.0 μg/mL) at final concentrations of 0.5, 1.0, 2.0, 4.0, or 6.0 μg/mL. Simultaneously, a positive control group was established by treating SZ95 cells with a mixture solution containing a final concentration of 3.0 μg/mL of 13‐*cis* RA [25] and 10.0 μg/mL of LA.

For the Nile Red staining groups treated with DHT or a mixture of DHT and TSS, the SZ95 cells were treated with 100 μL of DHT at a concentration of 20.0 μg/mL or with mixtures of DHT (20.0 μg/mL) and TSS at final concentrations of 0.5, 1.0, 2.0, 4.0, or 6.0 μg/mL. Concurrently, a positive control group was established by treating SZ95 cells with a mixed solution containing final concentrations of 3.0 μg/mL of 13‐*cis* RA and 20.0 μg/mL of DHT.

Following the above indicated treatment for 48 h, intracellular lipids in the SZ95 cells were quantified using Nile Red [26] staining. Specifically, 100 μL of DAPI dyeing solution (Beyotime Inc., Shanghai, China) aiming at staining the nuclei was added and incubated for 10 min at room temperature. After discarding the DAPI solution, 100 μL of Nile Red (10 μg/mL, PBS dissolution) was added to each well, and allowed to incubate at 37°C in dark environment for 20 min. The staining of cells was imaged under a fluorescence microscope (Leica Stellaris 5, Germany). At the same time, the emitted fluorescence intensities of Nile Red were detected at the excitation wavelength of 485 nm and the emission wavelength of 565 nm. The lipid synthesis ratio of SZ95 cells was calculated by referring to Formula (1‐2).

(1‐2) $$\text{Lipid synthesis ratio}\;\left(\%\right)=\left({\mathrm{FI}}_{T}/{\mathrm{FI}}_{B}\right)\times 100\%$$

where FI_T_ and FI_B_ are the average fluorescence intensities of the experimental group and the control group, respectively.

### 2.5 Triglyceride, Total Cholesterol, and Fatty Acid Quantification Assays

The SZ95 cells (1.5 × 10^5^ cells/mL, 2 mL per well) were seeded into six‐well plates and incubated with LA or the mixture of LA and TSS as referred to that of Section 2.4. The cell precipitate was collected by trypsin digestion and centrifugation. The cellular lipids were extracted by the addition of 500 μL of isopropyl alcohol to the pellet, which was subsequently homogenized while submerged in an ice bath. The obtained solution was centrifuged at 4°C at 12 000 rpm for 5 min, allowing for the collection of the supernatant for further analysis. The triglyceride, cholesterol, and fatty acid concentrations were measured using the triglyceride, cholesterol, and fatty acid quantitative analysis kits (Beyotime Inc.) according to the manufacturer's instructions. Standard curves were constructed using triglyceride, cholesterol, and palmitic acid standard solutions, respectively.

### 2.6 Western Blot Assay

The SZ95 cells (1.5 × 10^5^ cells/mL, 6 mL per bottle) were seeded into T25 bottles and incubated with LA (10 μg/mL) or the mixture of LA (10 μg/mL) and TSS (4.0 or 6.0 μg/mL) as shown in Section 2.4. After incubated for 48 h, the digestion process was made in the incubator using a trypsin digestion solution containing 0.25% EDTA for 2–3 min, followed by termination of the digestion with DMEM. The cell digestion solution was then centrifuged (1200 rpm/min for 5 min) to obtain precipitates by discarding the supernatant. After the protein was extracted, its concentration was detected using the BCA protein concentration assay kit (Beyotime Inc.). Using β‐actin as the internal reference protein, the related proteins AMPK, phosphorylated AMPK, mTOR, phosphorylated mTOR, Raptor, phosphorylated Raptor, SREBP‐1, FAS, and PPARγ were determined by western blotting. All antibodies were sourced from Shanghai Biyuntian Biotechnology Co., Ltd. Finally, imaging was performed with the Chemi DOC XRS+ gel imaging system (BIO‐RAD, Hercules, CA, USA).

### 2.7 Measurement of the mRNA Expression of SREBP‐1, FAS, and PPARγ

The cell culture/treatment procedures were the same as described in Section 2.6. The RNeasy RNA extraction kit (Beyotime Inc.) was used to extract the total RNA in SZ95 sebocytes. The BeyoFast SYBR Green One‐Step real‐time polymerase chain reaction (RT‐PCR) kit was adopted for conducting the RT fluorescent quantitative PCR measurement. The relative expression was normalized by $2^{-\Delta \Delta C_{T}}$ with GAPDH as an internal reference gene. The following primers were used in this study: SREBP‐1 (F: 5′‐CCTCCATGGGGTCAGTTGTC‐3′; R: 5′‐GACTTCTTGCAGGGAGACC‐3′); FAS (F: 5′‐CCCATGTCAATGAGCACAGC‐3′; R: 5′‐CAGTATGCCCACCACAAAGAG‐3′); PPARγ (F: 5′‐AGAGCCTTCCAACTCCCTCA‐3′; R: 5′‐GAAGAAACCCTTGCATCCTTC‐3′); GAPDH (F: 5′‐TCGGAGTCAACGGATTTGGT‐3′; R: 5′‐TTCCCGTTCTCAGCCTTGAC‐3′).

### 2.8 Immunofluorescence

The SZ95 cells (7 × 10^4^ cells /mL, 1 mL per dish) were seeded into laser confocal dishes for incubation with LA (10 μg/mL) or the mixture of LA and TSS (6.0 μg/mL). After incubated for 48 h and washed with PBS, the cells were fixed in the immunostaining fixing solution for 15 min. After washing, the immunostaining blocking buffer was added for blocking reaction at room temperature for 1 h. Thereafter, followed by the addition of the SREBP‐1 primary antibody for incubation at 4°C overnight, and incubation with the secondary antibody for 2 h. After washed and then stained with an Antifade Mounting Medium containing DAPI for staining the nuclei for 10 min, photographs were performed on a laser confocal microscope (Leica Stellaris 5). All reagents used for the immunofluorescence experiment were obtained from Shanghai Universal Biotech Company (Shanghai, China).

### 2.9 Statistical Analysis

The data analysis was carried out by GraphPad and data were expressed in the form of mean ± standard deviation. The comparison between groups was assessed through analysis of variance followed by the Tukey's test. The *p* value less than 0.05 was considered to be statistically significant.

## 3 Results

### 3.1 Characterization of TSS

**FIGURE 1:**
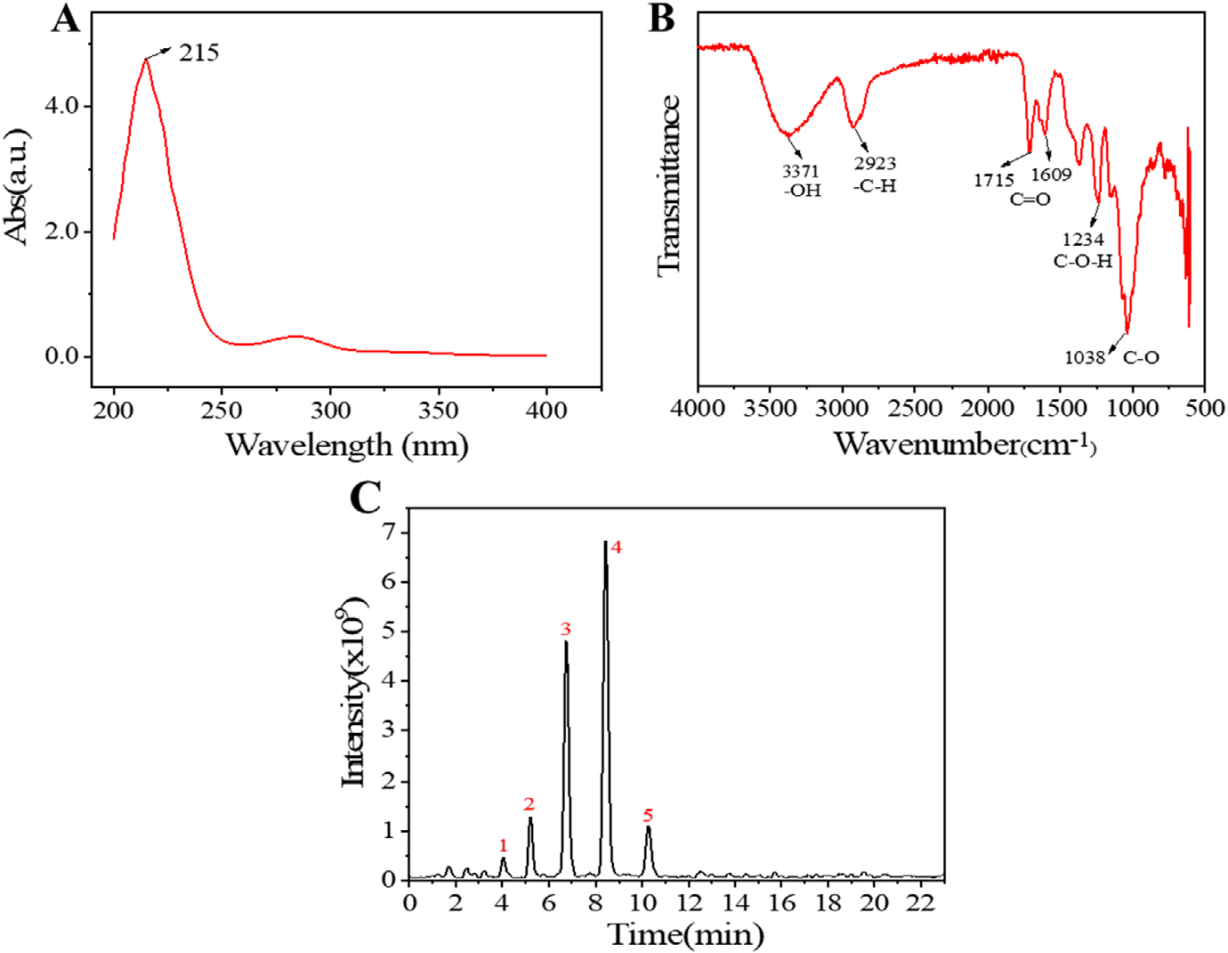
Composition and structure analysis for TSS: (A) UV–vis absorption and (B) FTIR spectra of the obtained TSS and (C) total ion flow diagram of TSS in UHPLC.

The extraction of TSS from tea seed meal using 70% ethanol was further extracted by petroleum ether and n‐butanol extraction, and finally purified by a D101 macroporous resin consisted column chromatography. The identification of the chemical structure and molecular formula of the saponins were through UV–vis absorption spectra, FTIR spectra and UHPLC–MS analysis. In the UV–vis absorption spectrum, a mainly characteristic absorption peak at 215 nm was observed (Figure 1A), indicating that saponins [27, 28] were the main components of the as‐obtained product. And a tiny absorption peak appeared at 280 nm might be resulted from the presence of a tiny amount of cinnamic acid or flavonoid [23, 29] impurities.

The obtained TSSF showed very similar FTIR spectra to that obtained by other researchers [23, 27, 29]. As shown in Figure 1B, the peaks at 3371 and 2923 cm^−1^ were ascribed to the –OH and C–H stretching vibrations, respectively. The stretching vibrations of the C=O bond was observed at 1715 and 1609 cm^−1^. While the peak at 1234 and 1038 cm^−1^ were the bending vibration peak in the C–O–H plane and the stretching vibration peak of the C–O–C, respectively.

**FIGURE 2:**
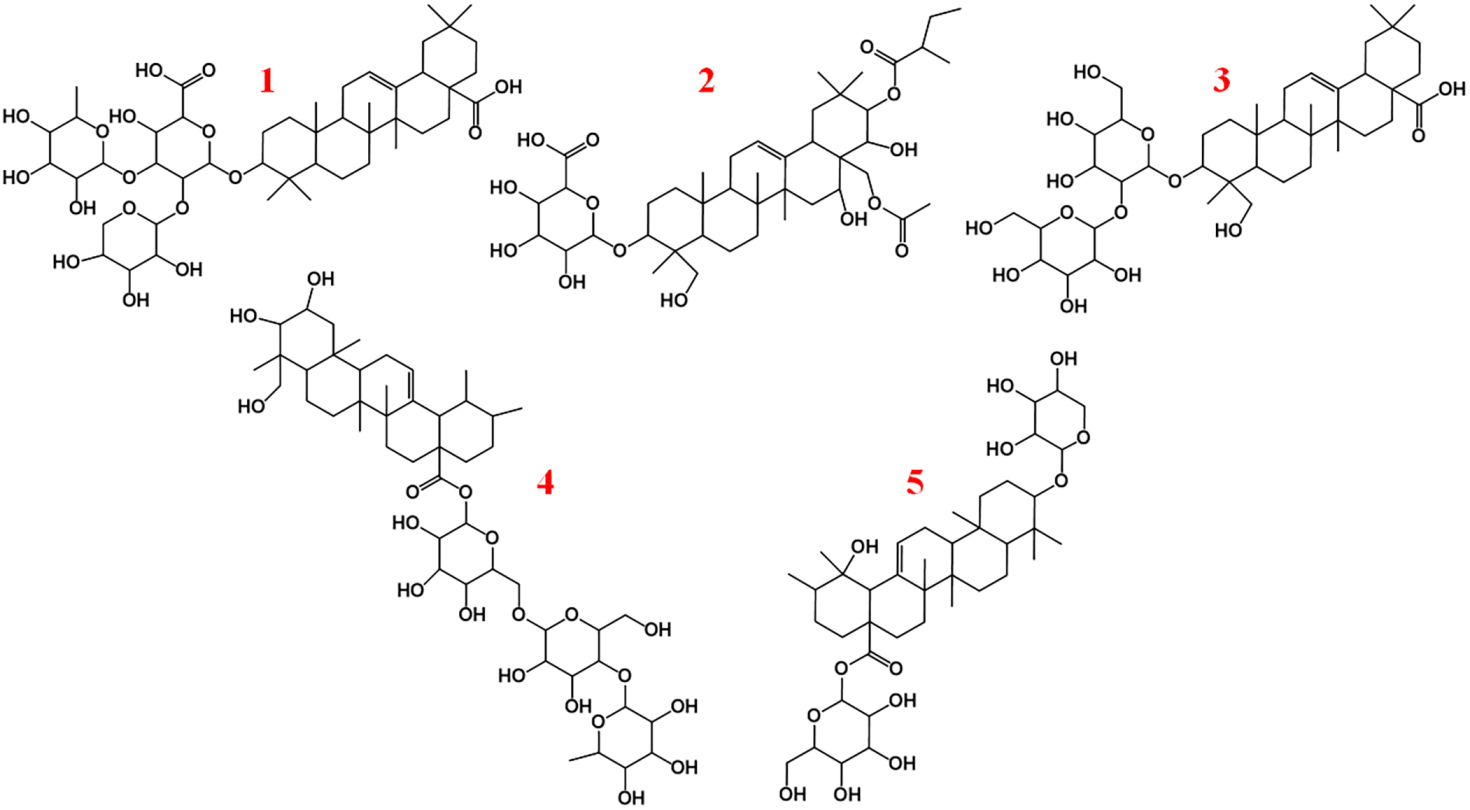
Detailed structure of the mainly five saponins.

The detailed structure of the obtained TSS was further analyzed by UPLC‐MS in a positive ionization mode. Five main peaks were obtained in the total ion chromatogram of TSS, as shown in Figure 1C. Table 1 sorted out the detailed information from that of UPLC‐MS. The fragment ions were compared and analyzed in MMSs‐public‐Expbioinsilico‐pos‐VS17 and MMSs‐pos‐Vanya‐Fiehn Natural Products Library. And through the website https://pubchem.ncbi.nlm.nih.gov/, we identified five kinds of saponins structure (the structure diagram was sorted out in Figure 2), and the main types of saponins were oleanic acid pentacyclic triterpene [30, 31, 32] with different glycosylation degrees and different types of glycogenin.

**TABLE 1:** UHPLC–MS analysis for the composition and structure of TSS.

Compound	Rt/(min)	[M + Na]^+^ (*m*/*z*)	Fragments (*m*/*z*)	Molecular formula
1	4.07	933.48	451;551;949	C_47_H_74_O_17_
2	5.21	831.45	215;405;505	C_43_H_68_O_14_
3	6.76	819.45	211;451;627	C_42_H_68_O_14_
4	8.44	981.50	405;505;699	C_48_H_78_O_19_
5	10.24	789.44	211;435;611	C_41_H_66_O_13_

### 3.2 Effects of TSS, LA, and DHT on Cell Viability

**FIGURE 3:**
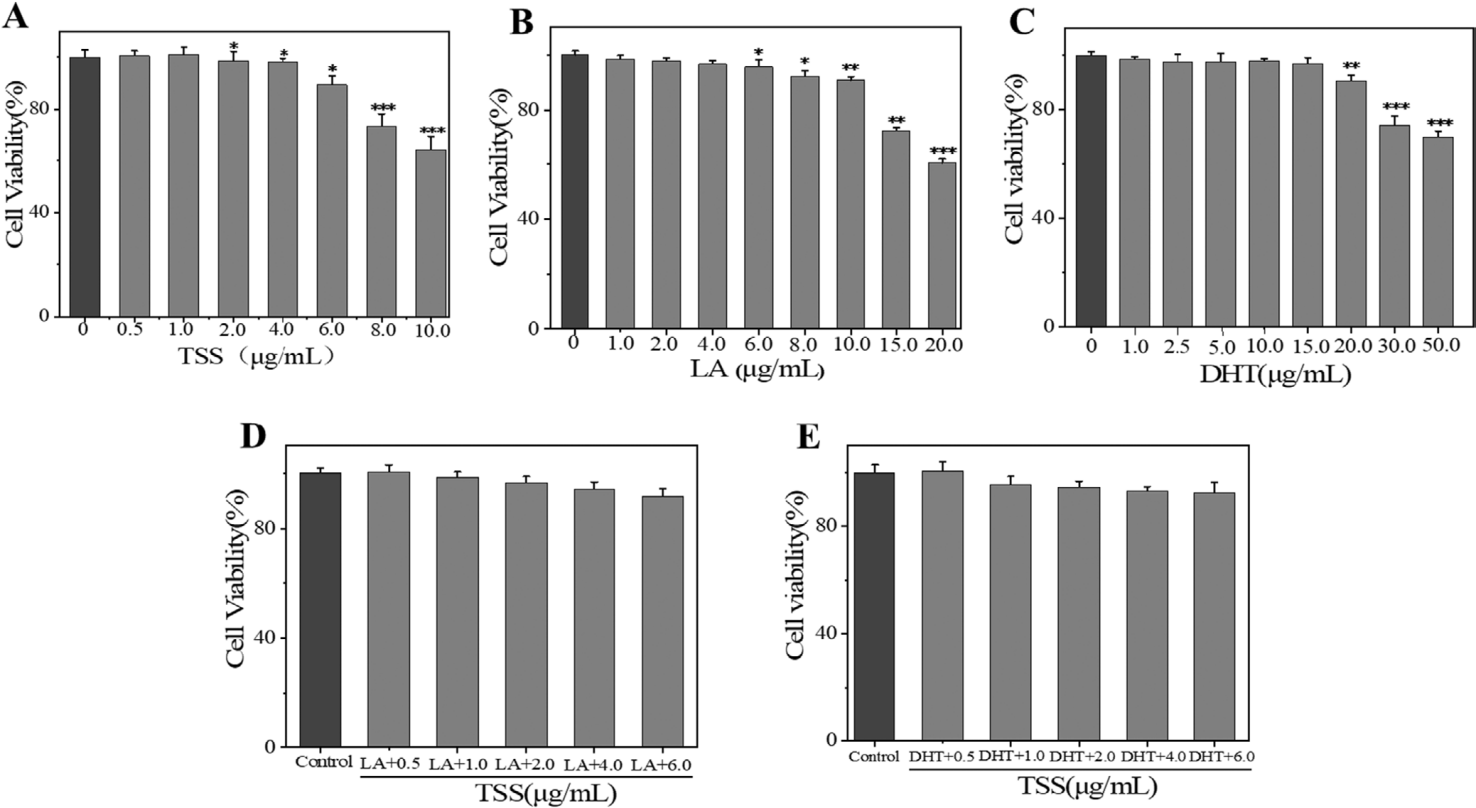
Effect of TSS, LA, and DHT on cell viabilities of SZ95 cells. Toxicity effects of (A) TSS, (B) LA, (C) DHT, (D) the mixture of TSS and LA, and (E) the mixture of TSS and DHT on SZ95 cells. Error bars indicated the standard deviation of three independent tests in triplicate (*, **, and *** indicated *p* < 0.05, 0.01, and 0.001 vs. control group).

To exclude the influence of TSS, LA, and DHT on cell viability, the concentration‐toxicity relations of these substances on SZ95 cells were determined by the MTT [33] method. The cell viabilities of SZ95 cells were greater than 90% at lower concentrations ranges of TSS from 0.5 to 6.0 μg/mL, and the cell survival ratios were less than 75% at higher concentrations of TSS from 8.0 to 10.0 μg/mL (Figure 3A). As shown in Figure 3B, the cell survival ratios of SZ95 cells were greater than 90% at lower concentrations of LA from 1.0 to 10.0 μg/mL, and the cell survival ratios were less than 75% at higher concentrations from 15.0 to 20.0 μg/mL. As shown in Figure 3C, cell viabilities remained above 90% at concentrations of DHT ranging from 1.0 to 20.0 μg/mL. When the concentration was in the range of 30.0–50.0 μg/mL, cell viabilities dropped below 75%. Meanwhile, the effects of co‐incubation of TSS with LA or DHT on cell viabilities were also investigated. After being treated with the mixture of 0.5–6.0 μg/mL of TSS and 10.0 μg/mL of LA or 20.0 μg/mL of DHT, the cell viabilities of SZ95 cells were still greater than 90% (Figure 3D,E). Therefore, we chosen the following indicated concentrations (0.5–6.0 μg/mL of TSS, 10.0 μg/mL of LA, or 20.0 μg/mL of DHT) to investigate the sebum‐control activities of TSS in the subsequent experiments.

### 3.3 TSS Suppresses LA‐ and DHT‐Induced Lipid Synthesis in SZ95 Sebocytes

**FIGURE 4:**
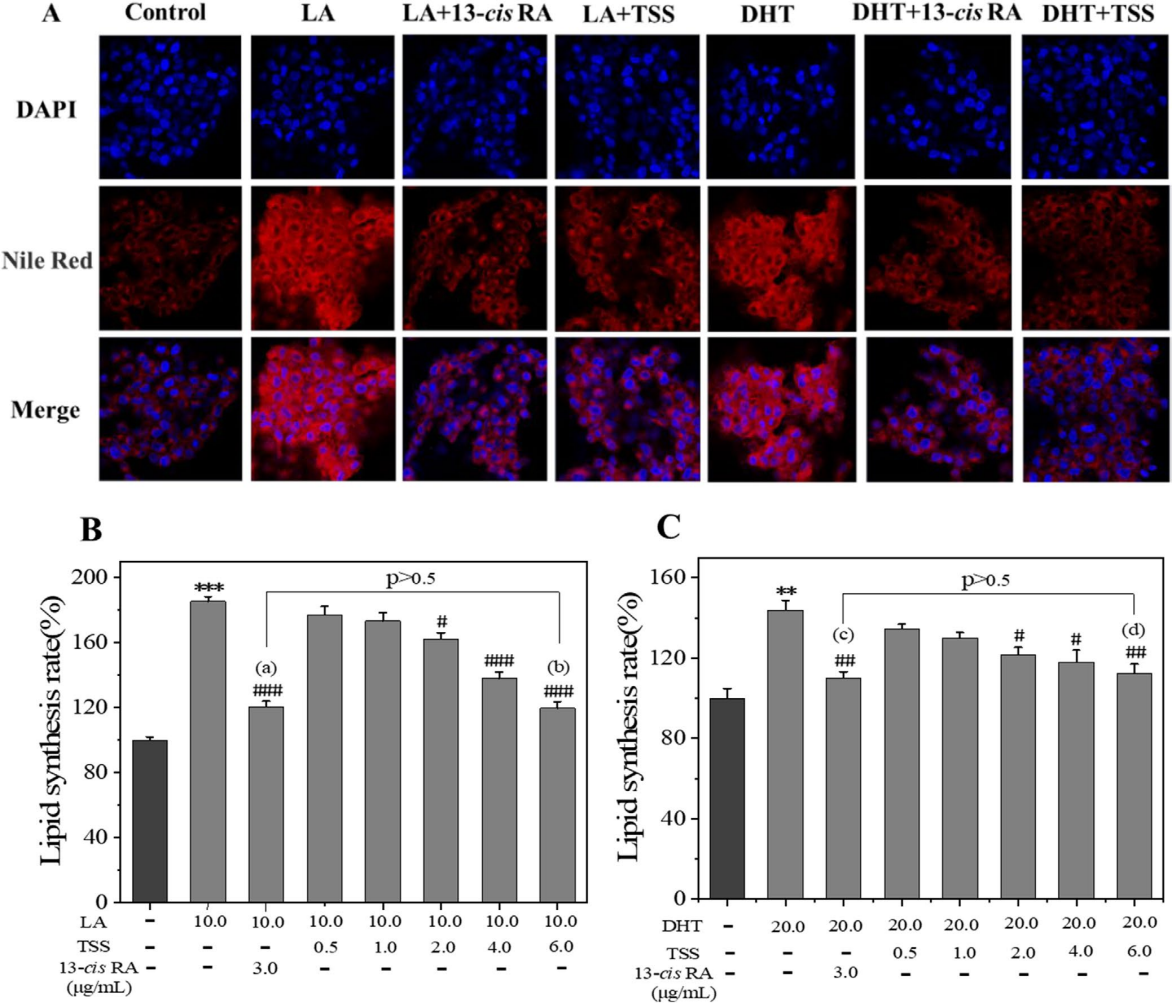
TSS decreased LA (10 μg/mL) or DHT (20 μg/mL) induced cellular lipid synthesis in SZ95 sebocytes. Intracellular lipids were stained by Nile Red (A) (the mass concentration of TSS was 6.0 μg/mL) and were further measured by the fluorescence intensities (B, C). Group (b) versus group (a), *p* > 0.5; Group (d) versus group (c), *p* > 0.5. Error bars indicated the standard deviation of three independent tests in triplicate (** and *** indicated *p* < 0.01 and 0.001 vs. control group; ^#^, ^##^, and ^###^ indicated *p* < 0.05, 0.01, and 0.001 vs. LA group or DHT group).

LA, an omega‐6 unsaturated fatty acid that can promote lipid production by inducing differentiation of SZ95 sebaceous cells and the differentiation is characterized by intracellular synthesis and accumulation of lipids [7], has been adopted as an inducer to stimulate sebum production. Consequently, we examined the impact of TSS on LA‐stimulated excessive lipogenesis in SZ95 sebocytes through the Nile Red staining method coupled with the fluorescence intensity measurement. The results revealed a markedly increase in the red fluorescence intensity of Nile Red in LA‐treated cells, indicating that LA induced overproduction of the lipids. However, co‐incubation of LA with 6.0 μg/mL of TSS led to a decreased fluorescence intensity (Figure 4A), suggesting the inhibited production of intracellular total lipids. The lipogenesis inhibition effect by TSS was centralization dependent, and the inhibition extent in lipid production for TSS (6.0 μg/mL) was similar as that of the positive control group using 13‐*cis* RA (3.0 μg/mL) (Figure 4B, *p* > 0.5), which meant that TSS had a similar effect to that of 13‐*cis* RA.

In addition, we also investigated the effects of TSS on hormones (such as DHT) induced lipogenesis, as revealed by the Nile Red staining and fluorescence intensity measurement. As observed, TSS decreased lipid production in a concentration‐dependent manner. And TSS (at 6.0 μg/mL) inhibited lipid production to a similar extent as the positive control group treated with 13‐*cis* RA at 3.0 μg/mL (Figure 4C, *p* > 0.5). These results indicated that TSS also held potential in curbing excessive lipogenesis induced by hormones.

**FIGURE 5:**
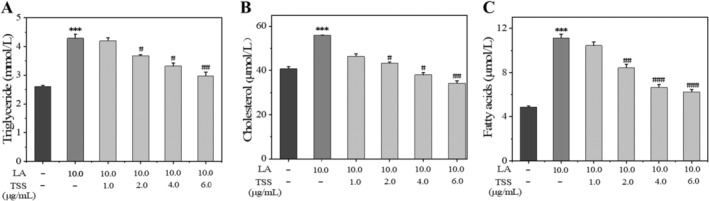
TSS decreased LA induced cellular lipid synthesis in SZ95 sebocytes. Triglyceride(A), cholesterol (B), and fatty acids (C) concentrations determined by commercially assay kits. Error bars indicated the standard deviation of three independent tests in triplicate (*** indicated *p* < 0.001 vs. control group; ^#^, ^##^, and ^###^ indicated *p* < 0.05, 0.01, and 0.001 vs. LA group).

Additionally, the introduction of LA caused an obvious increase in the synthesis of intracellular lipids, including triglycerides, cholesterol, and free fatty acids in the immortalized SZ95 sebocytes as measured by the commercial assay kits, while the treatment with TSS (at a concentration of 6.0 μg/mL) reduced the secretions of triglycerides by 50%, cholesterol by 53%, and free fatty acids by 100%, compared to the LA‐treated group (Figure 5A–C).

### 3.4 TSS Suppresses LA‐Induced Lipogenesis via the Activation of AMPK/mTOR Pathways

Because the underlying mechanism for TSS inhibited lipogenesis was not revealed, we attempted to clarify the signaling pathways that TSS inhibited sebocytic lipogenesis. Among the two important molecules regulating the metabolic balance of cells, the AMPK generally tunes catabolism while the mTOR mainly influences anabolism [34]. AMPK is a key regulator of mTORC1, an important protein complex consists of four core components of mTOR, Raptor, Deptor, and mLST8 [35], through the phosphorylation of mTOR and Raptor [9]. The activation of AMPK and suppression of mTOR are favorable for the inhibition of excessive lipid secretion.

**FIGURE 6:**
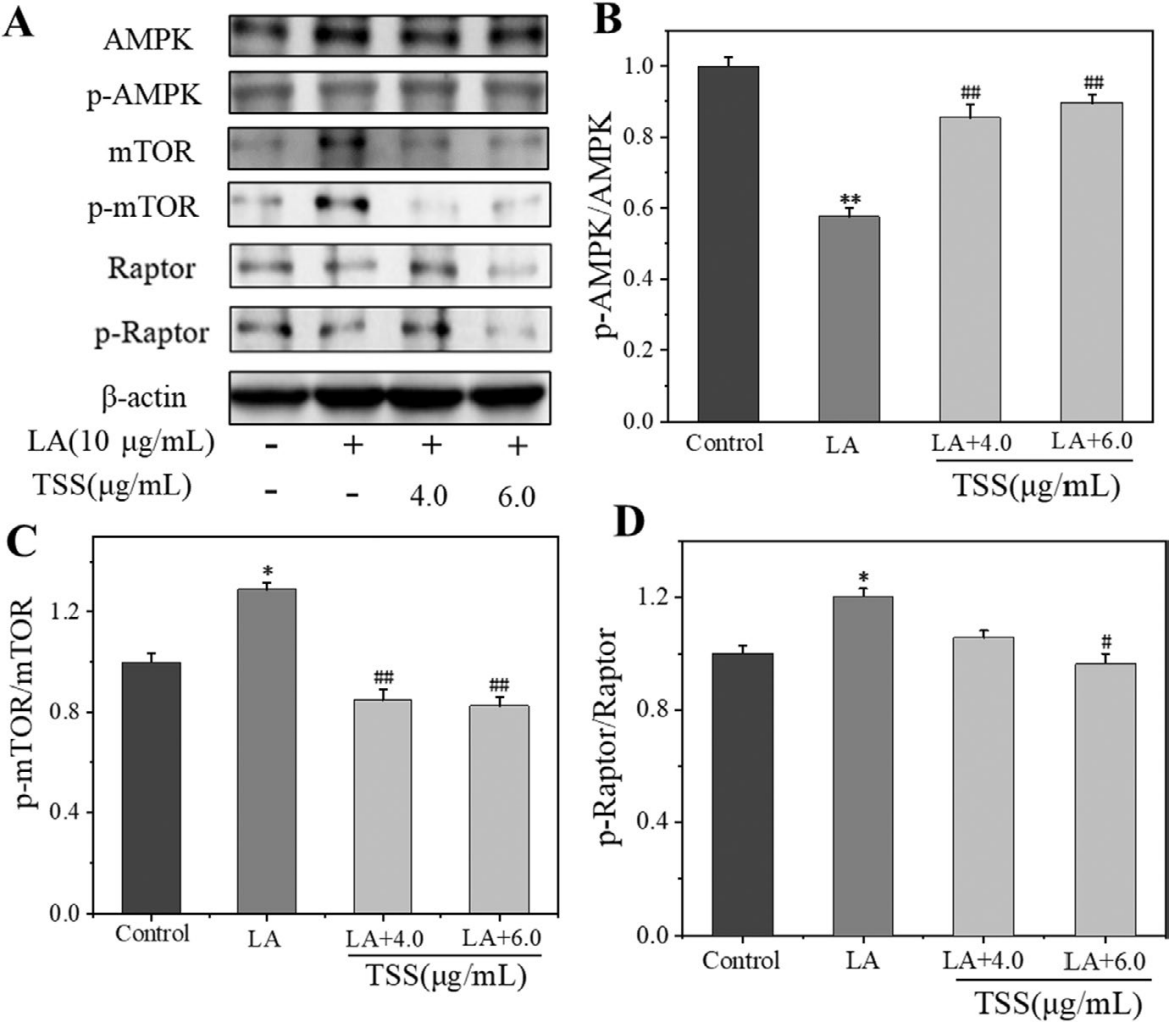
TSS changed the expression of proteins involved in the AMPK/mTOR pathway in LA‐stimulated SZ95 cells. (A) Protein bands from western blotting; (B–D) relative protein expression levels. Error bars indicated the standard deviation of three independent tests in triplicate (*, ** indicated *p* < 0.05, 0.01 vs. Control group, ^#^, ^##^ indicated *p* < 0.05, 0.01 vs. LA group).

The expression levels of AMPK, mTOR, and Raptor, as well as their phosphorylated products (p‐AMPK, p‐mTOR, and p‐Raptor), were probed by the western blot analysis. From Figure 6, we knew that the LA treatment for the SZ95 sebocytes significantly decreased the p‐AMPK/AMPK ratio but enhanced the p‐mTOR/mTOR and p‐Raptor/Raptor ratios. On the contrary, co‐incubation of LA with the TSS reversed this result. Compared with the LA group, TSS at a concentration of 6.0 μg/mL augmented the p‐AMPK/AMPK ratio by 32%, while decreased p‐mTOR/mTOR and p‐Raptor/Raptor ratios by 46% and 25%, respectively. These results indicated that the TSS activated the modulator of AMPK/mTOR, which mechanism was accounted for its anti‐lipogenesis effect.

Furthermore, the impact of TSS on the downstream regulatory targets of AMPK and mTOR were also investigated. As known, raptor modulates mTOR activation, which influences lipogenesis in sebaceous cells by regulating the expression of downstream transcription factors [11] including SREBP‐1 and PPARγ. As a member of the endoplasmic reticulum binding transcription factor family, SREBP‐1 is a major factor regulating lipid homeostasis through controlling its downstream lipogenesis synthesis enzyme of FAS for fatty acids synthesis [14]. PPARγ is a nuclear transcription factor associated with proliferation, differentiation and lipogenesis of sebaceous cells [36], and its expression is also regulated by AMPK.

**FIGURE 7:**
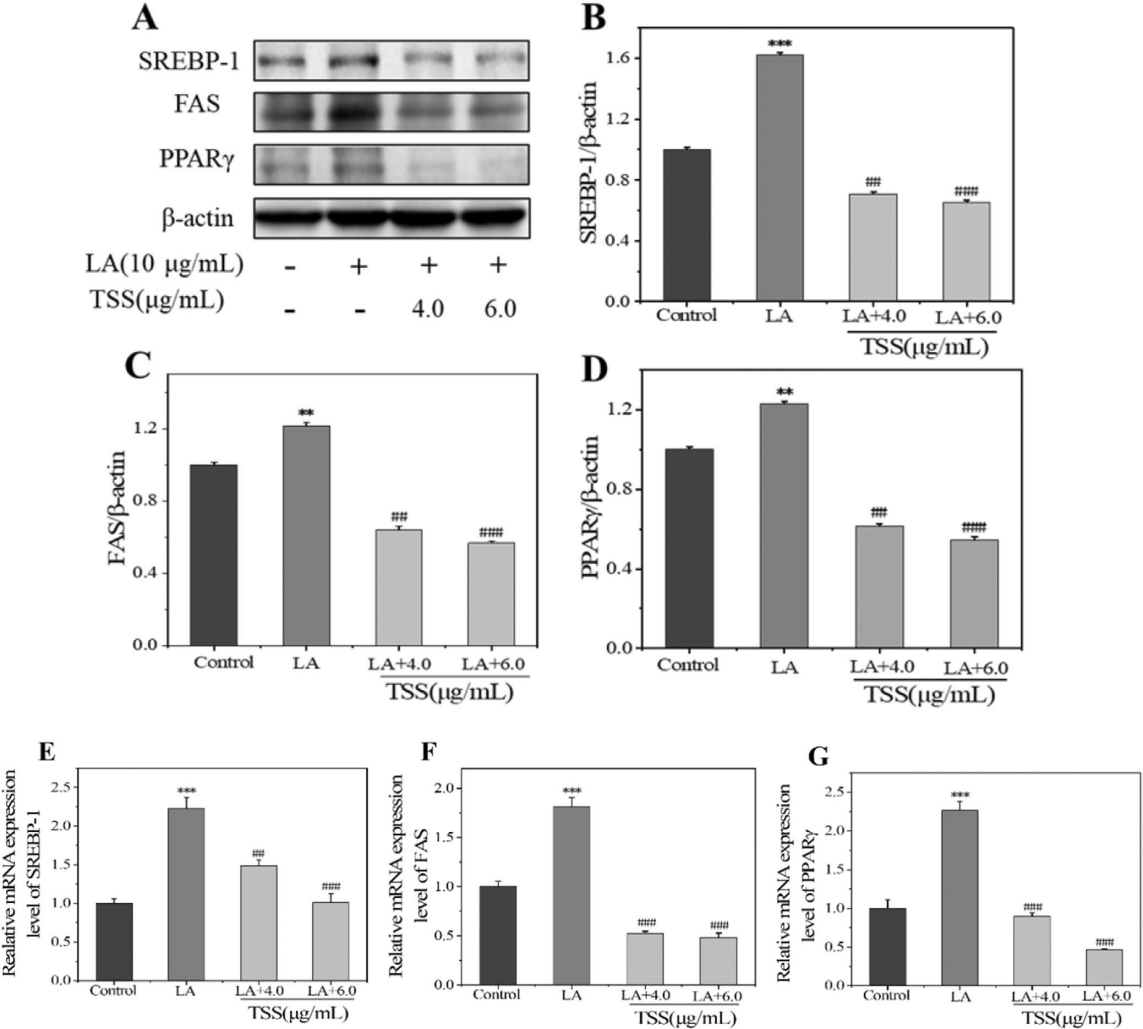
TSS inhibited the protein and mRNA expressions of SREBP‐1, FAS, and PPARγ in LA‐induced SZ95 cells. (A) Western blot protein bands. The same western‐blot bands for β‐actin were presented herein as that in Figure 6A because the measurements were all conducted in the same bath and adopted the same internal reference protein (i.e., β‐actin). (B–D) Relative protein expression levels; (E–G) relative mRNA expression levels. Error bars indicated the standard deviation of three independent tests in triplicate (**, *** indicated *p* < 0.01, 0.001 vs. Control group, ^##^, ^###^ indicated *p* < 0.01, 0.001 vs. LA group).

The protein expression levels of the transcription factors and enzyme, for example, SREBP‐1, PPARγ, and FAS, involved in sebocyte differentiation and lipid synthesis increased after treated by LA (Figure 7B–D). As expected, TSS obviously downregulated the expression levels of SREBP‐1, PPARγ, and FAS stimulated through LA by 96%, 65%, and 69%, respectively. These results suggested that TSS regulated the AMPK/mTOR pathway by suppressing the expression of downstream lipogenesis‐regulating transcription factors/enzyme, thereby reducing lipid synthesis in sebaceous cells.

Moreover, the mRNA expression levels of the SREBP‐1, FAS, and PPARγ were upregulated by the treatment with LA, but were downregulated following the co‐incubation with LA and TSS. And the decreased mRNA expression levels of the SREBP‐1, FAS, and PPARγ in SZ95 sebocytes presented a dose‐dependent manner (Figure 7E–G). The mRNA expression levels of SREBP‐1, FAS, and PPARγ for the 6.0 μg/mL TSS group were reduced by 120%, 130%, and 175%, respectively, relative to that of the LA group, which was also consistent with the trend of the western blot results for protein expression.

**FIGURE 8:**
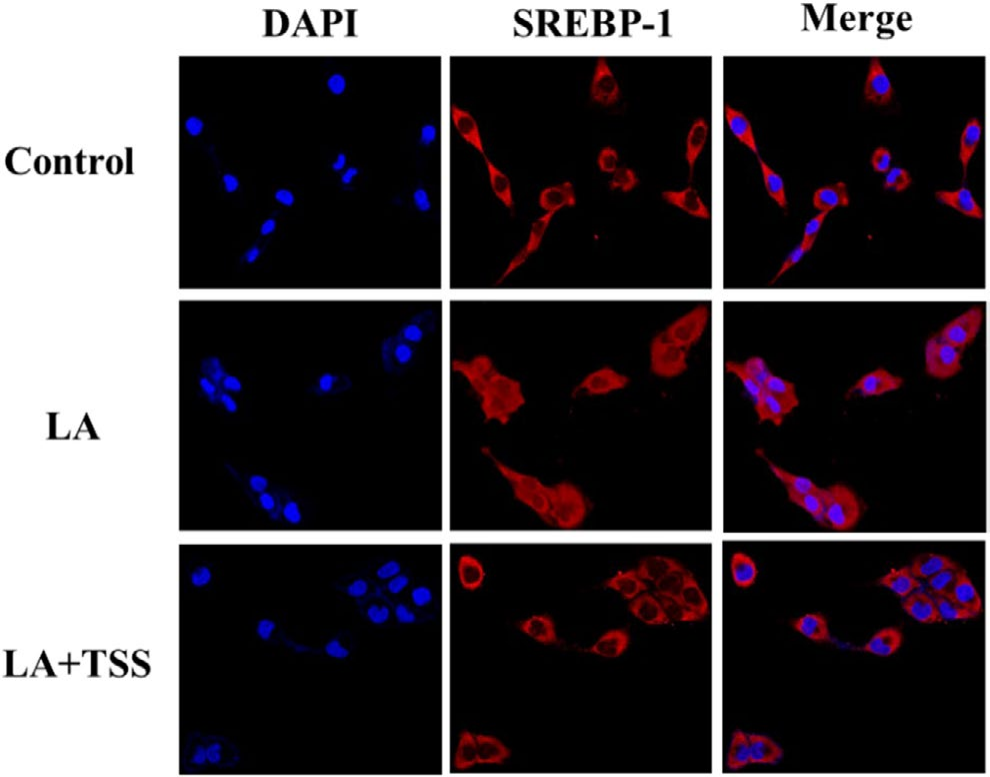
Effect of TSS on SREBP‐1 nuclear translocation in SZ95 cells induced by LA. The mass concentration of TSS was 6.0 μg/mL. After immunofluorescence staining, the localization of SREBP‐1 (red) was observed using confocal microscopy. The nucleus was stained with DAPI (blue). The results represented the results obtained from three separate tests.

SREBP‐1 itself exists in an inactive form in the cytoplasm and it can be activated after being cut by proteases and then transferred to the nucleus. The active domain of SREBP‐1 binds to the sterol response element in the promoter region of fat‐generating genes (such as FAS) to promote lipid synthesis [37, 38]. The results of immunofluorescence staining shown in Figure 8 indicated that the SREBP‐1 was mainly present in the cytoplasm for untreated group, but was transferred to the nucleus in large quantities after the LA incubation, indicating that the LA treatment activated SREBP‐1 for the generation of lipids. By contrast, the co‐incubation with 6.0 μg/mL of TSS inhibited the transfer of SREBP‐1 from cytoplasm to nuclear, thus disrupting the lipid synthesis.

## 4 Discussion

Excessive secretion of sebum will cause skin disorders such as oily skin, accompanied by destroyed water balance and annoying appearance of enlarged pores and dark skin symptoms. Botanic extracts are attractive as regulators of lipid metabolism because of their mildness, accessibility, safety, and biocompatibility. Saponins, as active components in plant extracts, possess the capability of enhancing lipid metabolism and reducing body fat [39]. The aim of this study was to investigate the potential effects of TSS on lipid metabolism in SZ95 sebocytes.

We first extracted crude saponins from tea seed meal using a 70% ethanol solution, followed by further purification using petroleum ether and *n*‐butanol. Finally, the crude saponins were purified by a D101 macroporous resin‐based column chromatography. In the following, the characteristic absorption peaks of TSS were confirmed by UV–vis absorption and FTIR spectra, which were in good agreement with the reports from literatures. Furthermore, the detailed structure of saponins in TSS was further determined by UHPLC–MS, and the obtained ion fragment was compared and analyzed with the database (MMSs‐public‐Expbioinsilico‐pos‐VS17 and MMSs‐pos‐Vanya‐Fiehn Natural Products Library). Through the website at https://pubchem.ncbi.nlm.nih.gov/, we identified five kinds of saponins structure that all belonging to the oleanic acid pentacyclic triterpenes.

DHT is a potent androgen that stimulates sebum secretion by binding to ARs located within sebaceous gland cells [40] and by activating the SREBP pathway [41]. And DHT is recognized as a key factor contributing to the development of acne [42]. Cheng et al. [43] demonstrated that treatment of SZ95 cells with DHT significantly increased the lipid production within these cells. Based upon these findings, we utilized DHT as a stimulant to establish a model of lipid overaccumulation in SZ95 cells and investigated whether TSS could inhibit lipid formation in these cells. The results indicated that 20 μg/mL of DHT markedly enhanced lipid synthesis in SZ95 cells, while TSS exhibited a concentration‐dependent reduction in lipid production. Notably, the inhibition of lipid production observed in the TSS (6.0 μg/mL) group was comparable to that seen in the positive control group treated with 13‐*cis* RA (3.0 μg/mL) (*p* > 0.5). This indicated that TSS can inhibit the generation of lipids in SZ95 cells induced by DHT, demonstrating its therapeutic potential in the treatment of acne.

Meanwhile, a model of excessive lipid accumulation in SZ95 cells was constructed using LA as the stimulus to investigate whether TSS can inhibit the lipogenesis in SZ95 sebocytes. The results showed that 10 μg/mL of LA significantly promoted lipid synthesis in SZ95 cells, and the lipid synthesis ratio was enhanced by 80% than the control group, which was revealed by the Nile Red staining and fluorescence measurement results. On the contrary, TSS decreased lipid synthesis in a concentration‐dependent manner, with a 60% decrease in lipid synthesis at 6.0 μg/mL compared to the LA group, and the extent of lipid production inhibition is similar to that of the positive control group using 13‐*cis* RA (3.0 μg/mL) (*p* > 0.5). Moreover, TSS at the concentration of 6.0 μg/mL reduced secretion of triglycerides, cholesterol, and free fatty acids by 50%, 53%, and 100%, respectively, in SZ95 cells when compared with that of the LA group.

AMPK and mTOR are two key intracellular signaling pathways that regulate lipid metabolism. There are some downstream transcription factors and enzymes of the AMPK/mTOR pathway that involve in the lipid synthesis: FAS is crucial in fatty acid synthesis, which is a downstream molecule of SREBP, a main transcription factor of most lipid synthases. PPARs are a family of orphan nuclear receptors that work by forming heterodimers with the retinoic acid X receptor in adipocyte differentiation and lipid synthesis [44]. Some studies have indicated that AMPK/mTOR and its downstream proteins play important roles in regulating lipid production in SZ95 cells [9, 11, 12, 14], and we would focus on studying the AMPK/mTOR pathway in TSS inhibited lipid accumulation.

**FIGURE 9:**
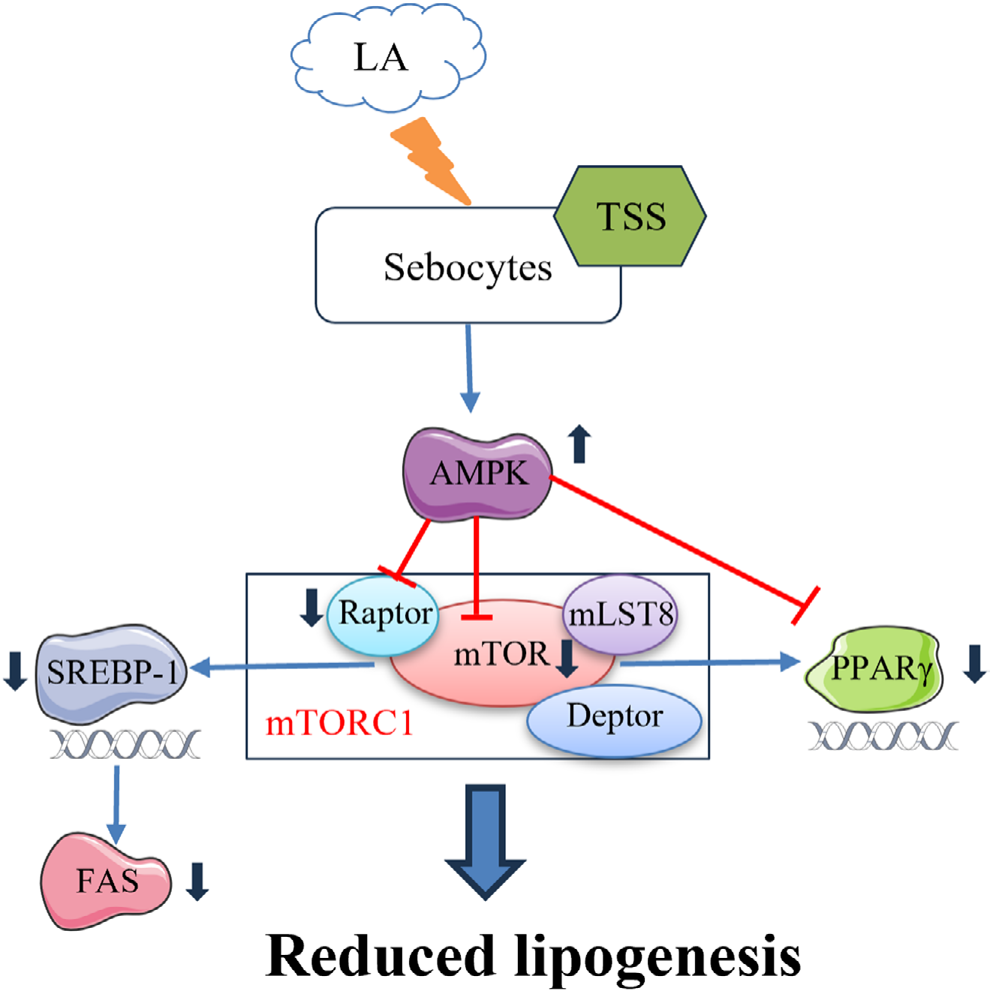
The underlying mechanism of inhibited lipogenesis by TSS in sebaceous SZ95 cells via regulating the AMPK/mTOR pathway.

Western blot results showed that LA at 10 μg/mL significantly suppressed p‐AMPK/AMPK ratio but increased p‐mTOR/mTOR and p‐Raptor/Raptor ratios. However, co‐incubation with LA and TSS reversed this result. Compared with the LA group, TSS at the concentration of 6.0 μg/mL increased p‐AMPK/AMPK ratio by 32%, while reducing p‐mTOR/mTOR and p‐Raptor/Raptor ratios by 46% and 25%, respectively. In addition, there was an obvious reduction in the protein expression levels of LA‐induced SREBP‐1, FAS, and PPARγ after treated by TSS (6.0 μg/mL), with the reduction extent by 96%, 65%, and 69%, respectively, relative to that of the LA group. Moreover, the mRNA expression levels of SREBP‐1, FAS, and PPARγ detected by RT‐PCR were upregulated by LA and downregulated by TSS. The mRNA expression levels of SREBP‐1, FAS, and PPARγ decreased by 120%, 130%, and 175%, respectively, in the presence of 6.0 μg/mL of TSS, when in contrast to that of the LA group. Moreover, TSS alleviated the transfer of SREBP‐1 from cytoplasm to nucleus. These results suggested that TSS inhibited the expression of downstream lipid‐producing transcription factors and enzyme by regulating the AMPK/mTOR pathway, thereby reducing lipid synthesis in sebaceous cells. Figure 9 illustrated the mechanism by which TSS inhibited LA‐stimulated lipid synthesis in SZ95 cells.

## 5 Conclusions

As a botanic extract, TSS was found to inhibit the excessive production of total lipids as well as triglyceride, cholesterol, and fatty acid in SZ95 sebocytes stimulated by LA. In addition, results elucidated that the TSS ameliorated lipogenesis largely through activating the AMPK/mTOR pathway and downregulated the protein and mRNA expression levels of downstream transcription factors/enzymes, such as SREBP‐1, FAS, and PPARγ. These results indicate that TSS holds promise as a sebosuppressive ingredients in cosmetics for treating excessive lipogenesis‐induced skin disorders. However, to incorporate TSS into commercial cosmetic products, more studies are needed, including in vivo efficacy evaluation, safety assessment, and formulation stability testing.

## Conflicts of Interest

The authors declare no conflicts of interest.

## Data Availability

The authors have nothing to report.
